# The Contributions of Prostate Cancer Stem Cells in Prostate Cancer Initiation and Metastasis

**DOI:** 10.3390/cancers11040434

**Published:** 2019-03-27

**Authors:** Wenjuan Mei, Xiaozeng Lin, Anil Kapoor, Yan Gu, Kuncheng Zhao, Damu Tang

**Affiliations:** 1 Department of Nephrology, the First Affiliated Hospital of Nanchang University, Nanchang 330006, China; wenjuanmei1986@gmail.com; 2 Department of Medicine, McMaster University, Hamilton, ON L8S 4K1, Canada; linx36@mcmaster.ca (X.L.); yangu0220@gmail.com (Y.G.); kunchengzhao@icloud.com (K.Z.); 3 The Research Institute of St Joe’s Hamilton, St Joseph’s Hospital, Hamilton, ON L8N 4A6, Canada; akapoor@mcmaster.ca; 4 Hamilton Urologic Oncology Research Center (HUORC), St Joseph’s Hospital, Hamilton, ON L8N 4A6, Canada; 5 The Hamilton Center for Kidney Research, St. Joseph’s Hospital, Hamilton, ON L8N 4A6, Canada; 6 Department of Surgery, McMaster University, Hamilton, ON L8S 4K1, Canada

**Keywords:** prostate cancer stem cells, prostate cancer initiation, metastasis, partial EMT

## Abstract

Research in the last decade has clearly revealed a critical role of prostate cancer stem cells (PCSCs) in prostate cancer (PC). Prostate stem cells (PSCs) reside in both basal and luminal layers, and are the target cells of oncogenic transformation, suggesting a role of PCSCs in PC initiation. Mutations in PTEN, TP53, and RB1 commonly occur in PC, particularly in metastasis and castration-resistant PC. The loss of PTEN together with Ras activation induces partial epithelial–mesenchymal transition (EMT), which is a major mechanism that confers plasticity to cancer stem cells (CSCs) and PCSCs, which contributes to metastasis. While PTEN inactivation leads to PC, it is not sufficient for metastasis, the loss of PTEN concurrently with the inactivation of both TP53 and RB1 empower lineage plasticity in PC cells, which substantially promotes PC metastasis and the conversion to PC adenocarcinoma to neuroendocrine PC (NEPC), demonstrating the essential function of TP53 and RB1 in the suppression of PCSCs. TP53 and RB1 suppress lineage plasticity through the inhibition of SOX2 expression. In this review, we will discuss the current evidence supporting a major role of PCSCs in PC initiation and metastasis, as well as the underlying mechanisms regulating PCSCs. These discussions will be developed along with the cancer stem cell (CSC) knowledge in other cancer types.

## 1. Introduction

Prostate cancer (PC) is the most common male malignancy and a major cause of cancer death in men in the developed world [1]. The prostate gland consists of two layers of epithelial cells, basal and luminal epithelial cells [2]. These two cell types have different properties; the majority of luminal epithelial cells express the androgen receptor (AR) and require AR signaling for survival, while basal epithelial cells are AR-negative, and thus not sensitive to castration [3]. As discovered by Charles Huggins [4,5], PC depends on AR signaling, and shares similarities with luminal epithelial cells. In this regard, human PC is widely viewed as being derived from luminal epithelial cells. Similar to other cancer types, PC displays heterogeneity. Among different cell types, a minor cell population is critical in PC formation, progression, and the formation of the heterogeneous PC mass. This unique cell population is defined as prostate cancer stem cells (PCSCs). In mice, PC can originate from both basal and luminal epithelial cells, indicating that PCSCs can be generated from either layer; this is consistent with the content of prostate stem cells (PSCs) in both layers [6].

The cancer stem cell (CSC) model states that CSCs are the driving force of cancer evolution and resistance to therapies. PC develops from high-grade prostatic intraepithelial neoplasia (HGPIN), which progresses to locally invasive carcinoma and then metastatic cancer [7,8]. PC predominantly metastasizes to the bone [9]. It was initially observed that surgical castration and estrogen injection resulted in significant tumor regression in 15 of 21 patients with metastatic PC (mPC) [4,5]; this discovery led to androgen deprivation therapy (ADT) as the standard treatment in patients with mPC. ADT showed remarkable response in more than 80% of patients; however, castration-resistant PCs (mCRPCs) commonly develop [10]. Although numerous treatment options are available for mCRPCs, these treatments can only marginally prolong the median overall survival (OS). Docetaxel-based therapy was reported to extend the median OS of CRPC for approximately three months [11,12], as resistance rapidly develops. The same trend also applies to the more potent second-generation anti-androgens approved by Food and Drug Administration (FDA) in 2011 and 2012 [13,14]. The steroid synthesis inhibitor abiraterone and AR antagonist enzalutamide showed only a four to five-month survival advantage in patients with taxane-resistant mCRPC [13,14]. To date, metastasis remains the overarching challenge in PC.

**Figure 1:**
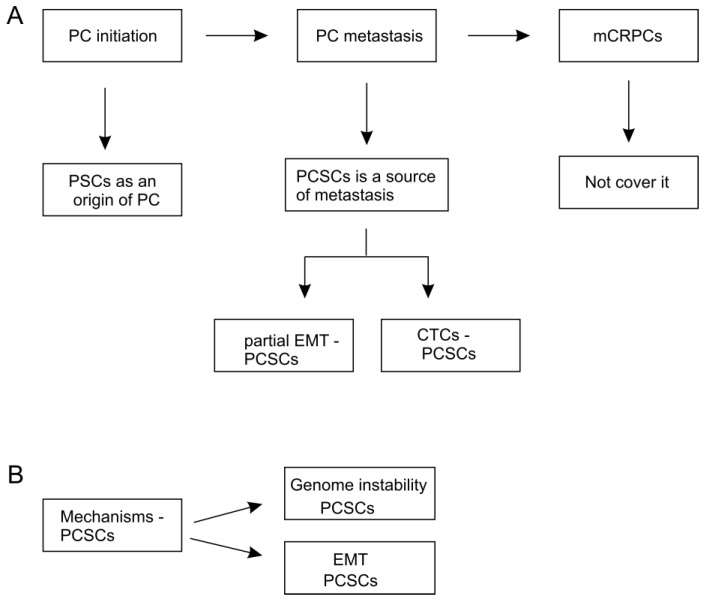
Review manuscript organization. (**A**) The typical prostate cancer (PC) pathogenesis involves tumor initiation, progression to metastatic diseases, and metastatic castration-resistant prostate cancers (mCRPCs) following androgen deprivation therapy (ADT) treatment. Prostate cancer stem cells (PCSCs) contribute to all these processes; this review will not cover the contributions of PCSCs to CRPC, but rather focus on the other two processes. PSC: prostate stem cells; CTCs: circulating tumor cells. (**B**) The mechanisms that regulate PCSC stemness will be discussed.

Mechanisms of PC initiation and metastasis have been extensively investigated. While our understanding remains incomplete, this colossal research effort clearly revealed complex mechanisms in play for PC initiation and metastasis. These mechanisms can be converged on regulating PCSC. In this review, we will briefly introduce PCSC, discuss evidence supporting PCSC being an origin of PC (Figure 1A), and review research for PCSCs playing critical roles in PC metastasis (Figure 1A). The underlying mechanisms, limitations, and future perspectives will be covered (Figure 1B).

## 2. Identification of PCSCs

The CSC concept was not novel, but it reinvigorated the field of cancer research following the isolation of CD44^+^CD24^−/low^ CSC from primary breast cancer in 2003 by Al-Hajj et al. [15], several years after the establishment of CD34^+^CD38^−^ CSC in acute myeloid leukemia (AML) in 1994 [16]. Since then, CSCs have been isolated in multiple solid cancer types [17,18], including bladder cancer [19], brain tumor [20], colon cancer [21,22], head and neck squamous cell carcinoma [23], liver cancer, lung cancer [24], melanoma [25], pancreatic cancer [26,27], and sarcoma [28]. The initial identification of solid tumor CSCs was largely based on the understanding that CSCs may express a set of surface biomarkers with their tissue stem cells (SCs) [29]. Human prostate SCs are α_2_β_1_^hi^CD133^+^ [30]; PCSCs were identified by Collins et al. in 2005 from primary human PC as CD44^+^α_2_β_1_^hi^CD133^+^ [31]. It was also reported that PCSCs derived from primary human PCs expressed the breast cancer resistance protein/BCPR (ABCG2) [32]. In both pioneer studies, CD44^+^α_2_β_1_^hi^CD133^+^ and BCPR^+^ PCSCSs were able to produce other types of PC cells, including those of AR^+^ [31,32]. Subsequently, Trop2^hi^, CD166/ALCAM, PSA^−/low^ (prostate-specific antigen), and ALDH1A1—with the latter being an intracellular protein—have been reported to define PCSCs or be associated with PCSC properties in primary PC [33,34,35,36].

PCSCs have also being identified in PC cell lines using cell surface markers and sphere formation assay. CD44^+^ cells from immortalized human prostate epithelial cells and xenograft tumors were more tumorigenic than their CD44^−^ counterparts, and exhibited upregulations in stemness genes, including OCT3/4, BMI1, and β-catenin; CD44^+^ cells were able to produce CD44^−^ PC cells [37]. In LNCaP cells, PSA^−/low^ cells displayed PCSC properties [36]. From DU145 cells, PCSCs have been isolated with sphere formation assays; these PCSCs can be maintained for at least 30 passage in 1.5 years without a clear decrease in sphere-forming capacity; the passage is facilitated by EGFR-mediated ERK activation [38]. More importantly, these PCSCs displayed a 100-fold increase in tumor initiation in NOD/SCID mice [39]. DU145 cell-derived PCSCs were CD44^+^ with an elevation in SOX2 expression [39,40]. Sphere formation was also used to enrich PCSCs from primary Gleason grades 3 to 5 PCs [41].

## 3. PCSCs as a Potential Origin of PC

The prostate consists of a basal and luminal layer of epithelial cells. To gain insights on PC pathogenesis, one of the key issues is the origin of PC. More relevantly, are PCSCs the origin? While there are no definitive answers, accumulating evidence seems to support this notion.

In AML, transformation of the hematopoietic stem cell causes the disease [42,43]. In solid tumors, the situation is much more complex. In the clinic, a lack of (basal) cells that are positive for the basal epithelial high molecular-weight cytokeratins (CKs) 34βE12 has been used as a diagnostic measure to confirm PC [44,45,46,47], as human PCs expressed the luminal profile of CKs [48], suggesting the luminal epithelial cells as the target cells for neoplastic transformation [49]. However, in view of PCSCs constituting a small percentage of PC cells and its AR-negative status, the observation that the vast majority of PC cells display luminal epithelial cell CKs does not exclude basal epithelial cells as a possible PC origin. In human prostate, PSCs with the surface antigens of α_2_β_1_^hi^CD133^+^ are located within the basal layer [30]. Impressively, human prostate epithelial cells could be isolated using CD49f^hi^Trop2^hi^; these basal epithelial cells, but not luminal epithelial cells, formed a prostate gland structure in the subcutaneous (s.c.) space of NOD/SCID/IL-2Rγ^null^ (NSG) mice, confirming the presence of PSCs in the basal layer [50]. Importantly, the ectopic expression of a cocktail of factors including activated AKT (myristoylated AKT), ERG, and AR only in the basal epithelial cells resulted in prostatic intraepithelial neoplasia (PIN) and PCs in NSG mice (Table 1) [50]. This report together with the basal layer location of the identified PCSC surface markers CD44^+^α_2_β_1_^hi^CD133^+^ and ABCG2 in the human prostate gland [31,32] support PSCSs being an origin of PC.

This concept is strengthened by investigations using mouse models for PC. By using a variety of surface antigens (Sca-l, CD49f, and Trop2) in combination with a regeneration assay, murine PSCs were detected in the basal layer [33,51,52]. Impressively, a single Lin^−^Sca-1^+^CD133^+^CD44^+^CD117^+^ basal cell was able to form prostate when implanted into the renal capsule space of nude mice [53]. Sca-1^+^ PSCs not only regenerated prostate glands with engrafts in the renal capsule of SCID mice, but also formed PIN lesions following the ectopic expression of AKT1 [54]. The concomitant expression of AKT and AR in Lin^−^Sca-1^+^CD49f^hi^ basal PSCs produced poorly differentiated PC in CB.17^SCID/SCID^ mice (Table 1) [55]; the activation of endogenous AKT by the deletion of PTEN caused an expansion of Lin^−^Sca-1^+^CD49f^hi^ basal PSCs, and these PSCs generated PCs following s.c. implantation into SCID mice (Table 1) [56,57]. The location of PCSs in the basal layer was also demonstrated using the lineage-tracing genetic approach [58,59,60]. Consistent with the above engraft-based studies, the specific deletion of PTEN in the basal epithelial cells was sufficient to induce PC [60,61]. Taken together, PSCs in the basal layer are able to initiate PC in response to PTEN–AKT oncogenic actions.

The luminal layer of the mouse prostate also contains PSCs. The concept of luminal PCSs is supported by the generation of prostate organoids using single luminal epithelial progenitors [62]. Lineage-tracing genetic approaches also revealed PSCs in both the basal and luminal layers, and that the deletion of PTEN in either layer of epithelial cells led to PC (Table 1) [63]. However, it was suggested that basal epithelial cells were more resistant to oncogenic transformation in vivo compared to the luminal epithelial cells [63], which is consistent with the observations that the luminal epithelial cell-originated tumors were more aggressive than those derived from basal epithelial cells [60]. In addition to PTEN deletion, lineage-tracing investigations using *Nkx3.1^+/^*^−^, *Hi-Myc*, and *TRAMP* transgenic mice support luminal epithelial cells being prone to PC origination, at least in murine models [64]. In a lineage-tracing effort, it was demonstrated that among the luminal epithelial cells of the mouse prostate, the Nkx3.1 expression cells can self-renew, reconstitute prostate ducts with renal capsule engraft, and initiate PC following PTEN knockout (Table 1) [65]. Additionally, genetically tracing the BMI1^+^ lineage of luminal epithelial cells revealed their resistance to castration; these cells possess abilities of self-renewal, tissue regeneration [66], and can generate PC upon PTEN deletion (Table 1) [67]. Interestingly, castration resulted in recurrent PCs (CRPCs) driven by BMI1^+^SOX2^+^ cells [67], implying an important role of SOX2 in conferring lineage plasticity in PCSCs. Both BMI1 and SOX2 are well demonstrated for stem cell maintenance and promoting PC [40,68,69,70,71]. Furthermore, in the mouse luminal layer, there exists a group of LY6D^+^ epithelial cells with resistance to castration, PSC capacities, and the ability to produce PIN lesions with PTEN-specific knockout in the cells (Table 1) [72]. Collectively, in approximately 10% of luminal cells resistant to castration, two different groups of PSCs, Nkx3.1^+^ and BMI1^+^ [66], along with LY6D^+^ PSCs, have been identified as origins for PC.

Thus, evidence supports the existence of luminal and basal stem cells in mouse prostate and its relationship to oncogenic signals (Table 1). For example, the PTEN–AKT axis is tumorigenic when they were directed in these PSCs [64]. However, in human prostate, only the basal epithelial cells are able to regenerate prostate gland structure and produce PC upon receiving ectopic oncogenic signals [50]. These discrepancies may be a result of the unique differences in the pathological process between humans and mice. Nonetheless, it was observed that tumors that originated from human basal prostate epithelial cells can be maintained by the luminal cancerous cells of PCSC with SOX2 upregulation [73], suggesting a lineage switch during PC progression. This concept is consistent with the plasticity of SCs and CSCs [74,75], and also suggests that CSCs are evolving during the course of cancer progression. Evidence supporting the evolution of CSCs includes the general intratumoral heterogeneity across multiple tumor types [76,77], the generation of xenograft tumors with different properties from a single lineage [78], and the genomic instability associated with CSCs [79]. Collectively, accumulative evidence suggests a model that alterations in PSCs result in PCSCs that initiate PC. This model is supported by the Lgr5^+^ intestine stem cells as an origin of colorectal cancer [80]; glioblastoma requires tissue stem cells, and the ablation of Nestin^+^ CSCs caused glioblastoma regression [81].

## 4. PCSCs as a Source of PC Metastasis

Metastasis accounts for more than 90% of cancer-associated deaths [82,83], and remains the pressing challenge in cancer research. Metastasis is an inefficient process, as it requires the completion of multiple key steps [84]. Tumor cells are disseminated, and enter the blood stream through intravasation, which is a process facilitated by angiogenesis; in the circulation, cancer cells manage to survive and cross the vessel walls into the target organ (extravasation). From there, some cancer cells survive the foreign environment and initiate secondary tumor formation (colonization) [85,86]. Leaving the primary site and arriving at the secondary organs require epithelial cancerous cells to undergo epithelial–mesenchymal transition (EMT) [87,88]. To grow into metastatic tumors, cancerous cells reverse back to their epithelial status through mesenchymal–epithelial transition (MET) [89,90]. These sequential transitions between EMT (dedifferentiation) and MET (differentiation) are powered by cell plasticity, which is an essential property of CSCs. Additionally, cancerous cells at a foreign site need to be able to initiate tumors, just as in the primary site, in which CSCs are an origin. Collectively, evidence favors an important role of CSC in cancer metastasis [91,92].

### 4.1. The Contributions of EMT in PCSCs-Participated Metastasis

EMT plays a major role in regulating CSC properties [93], and is critical in cancer cell dissemination and its travel to metastatic sites [87,88]. By tracing the lineage of mouse pancreatic epithelial cells with KRasG12D expression and p53 knockout, cells with EMT were detected in circulation, which possessed stem cell properties and were able to seed in the liver [94], providing in vivo evidence for EMT promoting the dissemination of cancer cells and their seeding in a metastatic organ. Lineage-tracing mouse models with oncogenic signals directly targeting a particular type of epithelial cells also demonstrated a critical role of EMT in the metastasis of colon cancer, breast cancer, skin squamous cell carcinoma, and prostate cancer [95]. Using mice with prostate-specific *PTEN*^−/−^ and KRasG12D expression together with a vimentin–GFP reporter, Ruscetti et al. reported epithelial tumor cells (EpCAM^+^/Vim-GFP^−^), hybrid EMT (EpCAM^+^/Vim-GFP^+^) tumor cells, and mesenchymal-like (EpCAM^−^/Vim-GFP^+^) tumor cells [95,96] which likely resulted from a full EMT transition [95]. Both EpCAM^+^/Vim-GFP^+^ and EpCAM^−^/Vim-GFP^+^ tumor cells were detected in circulation, exhibited stemness properties, and were much more invasive compared to epithelial tumor cells [96]. Interestingly, the hybrid EMT (EpCAM^+^/Vim-GFP^+^) tumor cells but not mesenchymal-like (EpCAM^−^/Vim-GFP^+^) cells initiated metastatic tumor growth in the lung [96]; these observations indicated that the hybrid EMT PC cells possess lineage plasticity, which mediates MET transition and the subsequent formation of macrometastasis. Similar observations were also reported in hair follicle (HF)-derived squamous cell carcinoma (HF-SCC); hybrid EMT HF-SCCs were more plastic and more aggressive than HF-SCC with full EMT transition [95,97,98]. Furthermore, the existence of CTC breast cancer cells with partial EMT was also demonstrated [99]. In a small cohort of 81 PC patients, partial EMT CTCs with the antigen profile of CK^+^/Vim^+^/CD45^−^ were associated with metastasis [100].

At the center of EMT is a set of core transcription factors (TF) that execute EMT, including SNAIL, TWIST, and ZEB [87]. In human mammary epithelial cells (HMECs), the ectopic expression of either TWIST1 or SNAIL induced EMT; the resultant HMECs displayed properties of breast epithelial stem cells in terms of expressing the antigen profile of CD44^high^CD24^low^, forming mammospheres, and a generating mammary gland structure in immunocompromised mice [101]. In either HER2 or HRASV12 transformed HMECs, the enforced expression of TWIST or ZEB induced breast cancer stem cells (BSCSs) [101]. The association of BCSCs with EMT was also observed in HMECs transformed with the combination of hTERT, SV40 T/t, and HRASV12 [102]. In the mouse model of KRAS and p53 for pancreatic cancer, the deletion of ZEB1 inhibited stemness and metastasis [103]. In PC, evidence suggests TWIST1 contributing to bone metastasis [104]; TWIST1 enhances PC metastasis by upregulating HOXA9 through affecting WDR5 expression and chromatin structure [105,106]. The upregulation of histone methyltransferase, MMSET/WHSC1, and p53 mutation induce EMT in PC through the activation of TWIST1 [107,108]. Recent developments reveal a central role of ZEB1 in promoting EMT, CSC, and metastasis [109]. It has been recently reported that the ZEB1–miR375–YAP1 pathway promotes PC metastasis via regulating EMT and MET; while ZEB1 induces EMT, miR375 initiates MET via the inhibition of YAP1 expression in PC [110]. ZEB1 was reported to collaborate with the SRC kinase to drive EMT in vitro. In human PC metastases (*n* = 185) and bone xenografts, ZEB1 and E-cadherin were both expressed at high levels, suggesting the occurrence of MET [111]. Collectively, functional and clinical investigations strongly support a critical role of partial EMT in CSC and PCSCs in the case of PC-derived metastasis.

### 4.2. PCSCs in Circulating Tumor Cells (CTC) Lead to Metastasis

Similar to the first demonstration of CSC following the transplantation of a single leukemia cell into mice in 1937 [112], circulating tumor cells have been known for 150 years since their first report in 1869 by Ashworth [113]. However, their importance in tumorigenesis has only been recently realized as a result of knowledge and technological advances. The involvement of the vascular pathways in metastatic spread and the “seed and soil” hypothesis of Stephen Paget [114] highlighted circulating tumor cells (CTC) as “seeds” or source of distant metastasis [85,86,115]. CTCs were particularly enriched in patients with metastatic disease of PC, breast cancer, ovarian cancer, colorectal cancer, lung cancer, and other cancers [116], and were associated with reductions in overall survival in patients with breast, colorectal, and lung cancers [117,118,119]. CTCs possess a significant prognostic value in diagnosis for PC metastasis [120,121]. The EpCAM cell surface marker-based CellSearch platform has been approved by the FDA (Food and Drug Administration) for prognostic diagnosis in patients with breast, prostate, and colorectal cancers [122].

In line with recently identified intratumoral heterogeneity [76,77], CTCs also consist of multiple types of cancer cells [123]. Thus, it is conceivable for the CSC population in CTCs to be metastasis-initiating cells [124,125]. The existence of this CTC–CSC population is supported by the identification of breast cancer CTCs undergoing EMT [99], which is a process playing a critical role in CSCs. A subpopulation of breast cancer CTCs marked with EpCAM^+^CD44^+^CD47^+^MET^+^ was enriched with metastasis-initiating capacity and associated with a reduction in overall survival (Table 2) [126]. A subgroup of CTCs from breast cancer patients with brain metastasis were isolated, cultured, and selected for brain metastasis markers EpCAM^−^HER2^+^EGFR^+^HPSE^+^NOTCH1^+^; when compared to unselected CTC lines, these CTCs were capable of forming brain and lung metastases in nude mice (Table 2) [127]. The observation that these CTCs were EpCAM^−^ suggests that they have EMT properties. CTCs’ lines with CSC propensities were also demonstrated for colorectal cancer; these CTCs were able to generate xenografts after subcutaneous injection and liver metastasis following intrasplenic injection (Table 2) [128]. In PC, EGFR^+^ CTCs were suggested to be involved in bone metastasis [129]; EGFR mediates SOX2 expression in PCSCs [40]. CTCs with partial EMT were associated with PC metastasis; these CTCs were CK^+^Vimentin^+^CD45^−^ (Table 2) or EpCAM^+^CK^+^E-cadherin^+^Vimentin^+^N-cadherin^+^O-cadherin^+^CD133^+^ [100,130]. Collectively, while definitive and directive evidence for the existence of CTC PCSC and the role of CSCs as “seeds” for distant metastasis is currently lacking, the growing evidence obtained from multiple domains nonetheless supports CTC–CSCs playing a pivotal role in distant metastasis (Table 2).

### 4.3. Association of PCSCs with Metastasis

Evidence reveals a relationship between CSCs and metastasis in general [131] as well as between PCSCs and PC metastasis [132]. Breast cancer CSCs isolated from primary tumors produced metastasis upon implantation into the mammary fat pad of NOD/SCID mice [133]. In colorectal cancer, CD26^+^ CSCs generated liver metastases following implantation into the mouse cecal wall [134]; in pancreatic cancer, CD133^+^CXCR4^+^ CSCs were responsible for metastasis [26] and the overexpression of CXCR4 enhanced the metastatic potential of pancreatic cancer cells [135]. The stromal-derived factor 1 (SDF-1)/CXCL12 and its chemokine receptor CXCR4 play a critical role in the retention or homing of hematopoietic stem cells in bone marrow [136,137], suggesting CXCR4 playing a role in “seeding” cancerous cells for metastasis. In this regard, the SDF-1/CXCR4 pathway plays a critical role in the interaction between the tumor and its microenvironment [138,139]. CXCR4 expression is associated with the metastasis of human non-small cell lung cancer (NSCLC) [140], and contributes to breast cancer metastasis [141]. The SDF-1/CXCR4 axis directs PC metastasis to the bone [142], which is consistent with a critical role of SDF-1/CXCR4 in bone metastasis [143]. SDF-1 in PC stromal fibroblasts can be induced through the recruitment of mesenchymal stem cells (MSCs) [144], and human PC was reported to contain cells with the properties of MSCs [145]. In hTERT-immortalized human prostate epithelial cells, CD133^+^ cells displayed stemness along with an elevation in CXCR4 expression [146]; similarly, NANOG induced CSC propensities in PC cells concurrently with the upregulation of CD133 and CXCR4 [147]. The CXCR4 ligand CXCL12γ was reported to induce PCSCs and thereby promote PC metastasis [148]. Taken together, accumulative evidence supports a role of CSCs in general and PCSCs in particular as “seeds” of metastasis, in part via the SDF-1/CXCR4 axis. Of note, SDF-1 is expressed at high levels in lymph nodes, lung, liver, and bone marrow, which are the common organs of metastasis [149].

Additionally, functional evidence supports a critical role of PCSCs in PC metastasis. MicroRNA-141 was reported to suppress PCSC properties of CD44^hi^ PC3 and CD44^+^ DU145 cells and the cell’s ability to generate lung metastasis in an orthotopic PC model using NOD/SCID mice; the downregulation of microRNA-141 was observed in CD44+ primary PCs [150]. Likewise, miR-34a possesses similar functions in the suppression of PCSCs and thereby PC metastasis [151].

## 5. Pathways Regulating CSCs

In addition to cancer initiation, CSCs are widely regarded to play a central role in cancer progression, particularly in metastasis and resistance to therapies; in terms of PC, evidence supports PCSCs being critical in PC metastasis (this review) and CRPC development [152]. Solid tumors are not only heterogeneous but also have extensive intratumor heterogeneity [76,77]. The proportion of cells with CSC properties increases with cancer progression [153]. In this regard, CSCs including PCSCSs are heterogeneous and regulated by complex mechanisms; at the center of these regulations is the possession of CSCs’ plasticity.

### 5.1. Dynamically Maintaining CSC “Stemness”

**Figure 2:**
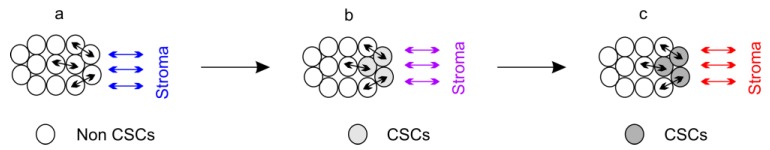
A dynamic model of cancer stem cells (CSCs). Intratumoral communications (black arrows with double directions) and tumor stromal communications (colored arrows with double directions) in (**a**) lead to the generation of CSCs (**b**). These communications will also drive CSC evolution (**c**).

CSCs are commonly associated with a specific set of surface antigens, such as CD34^+^CD38^−^ for AML, CD133^+^, CD44^+^, and other antigens for solid tumors, based on their ability to initiate tumors in nude and NOD/SCID mice [18]. However, CSCs may not inclusively reside in cells marked by these markers. For incidence, with the NOD/SCID/IL2Rγ^−/−^ (NSG) mouse line that is more receptive to xenograft formation, CD34^+^CD38^+^ AML cells were also able to initiate tumors [154]. Similarly, CD133^+^ NSCLS cells were substantially more efficient at producing xenograft in NOD/SCID mice [24]; in NOD/SCID/IL2Rγ^−/−^ mice, CD133^+^ and CD133^−^ NSCLC cells generated xenografts at rates of 6/11 and 7/13, respectively [155]. While CD133^+^ marks brain CSCs [20], both CD133^+^ and CD133^−^ glioblastoma cells displayed CSC properties and generated xenografts in immunocompromised mice with comparable efficiencies [156]. Human PCSCs were identified as CD44^+^α_2_β_1_^hi^CD133^+^ [31] and CD133^+^ cells isolated from 22Rv1 human PC cells were enriched with a set of CSC genes (CD44, OCT4, c-MYC, and BMI1) [157]; in a set of hTERT-immortalized human primary prostate cells, CD133^+^ and CD133^−^ cells comparably produced xenografts in NOD/SCID mice [158]. A difference in PCSC-associated proteins was also observed in aldehyde dehydrogenase (ALDH), which is a signature protein of CSC [159,160]. ALDH1A1 has been suggested to be associated with PCSCs [161,162,163,164]; a significant upregulation of ALDH3A1 in PCSCs and following PC progression was also reported [165]. In mouse models for PC, both the basal and luminal epithelial cells are able to initiate tumors (see Section 3). Intriguingly, tumors generated from human basal prostate epithelial cells were maintained through luminal cancerous cell-derived PCSCs [73]. The variations discussed above suggest communications between different populations of CSCs; alternatively, CSCs may be maintained via a dynamic manner depending on intratumoral and environmental cues (Figure 2).

The latter model is different from the classic concept of CSCs by emphasizing CSCs as a status of regulation rather than a subpopulation of cells that are intrinsically wired. This model may explain the isolation of CSCs from numerous cancer cell lines despite having been cultured for decades in 10% serum, which is a condition that is not supportive for CSCs. For incidence, DU145-derived spheres were 100-fold more tumorigenic in NOD/SCID mice compared to monolayer cells; in the presence of 10% serum, sphere cells proliferated significantly slower [39]. If what was preserved is in fact a CSC status instead of CSCs, this may explain the isolation of CSCs from cancer cell lines.

Intriguingly, this model of CSCs has recently gained in vivo support [166]. In a mouse model for colorectal cancer with the Lgr5 promoter driving the expression of diphtheria toxin receptor (DTR) to allow the controllable ablation of Lgr5^+^ cells, de Sousa e Melo et al. reported that the selective ablation of Lgr5^+^ CSCs with the addition of diphtheria toxin stopped tumor growth without tumor regression [167,168]. The removal of diphtheria toxin regenerated Lgr5^+^ CSCs and renewed tumor growth [168]. The depletion of Lgr5^+^ CSCs substantially suppressed liver metastasis [168], supporting the importance of CSCs in distant metastasis. Impressively, the diphtheria toxin-induced ablation of Lgr5^+^ CSCs in liver metastases resulted in tumor regression, and the growth of metastatic tumors was not renewed after the administration of diphtheria toxin was stopped [168]. This provides a plausible scenario to therapeutically target CSCs in colorectal cancer metastases. The research by de Sousa e Melo et al. [168] provided the first in vivo evidence suggesting that the stemness of CSCs, at least in primary colorectal cancer, can be acquired by dedifferentiation from cancer cells through unknown mechanisms, and the acquisition of CSCs ensures tumor growth and thus possible progression.

### 5.2. Mechanisms Regulating CSC Plasticity

CSC plasticity is regulated by complex mechanisms. External cues include interactions with stromal cells, hypoxia, and inflammation [92]. The contribution of hypoxia to CSCs is consistent with the hypoxic conditions observed for many adult SC niches [169]. Just as stem cells show properties of immune privilege [170], CSCs also possess these propensities [171]. These external factors will work through internal mechanisms or pathways for CSC regulation.

CSC plasticity is regulated by genome instability [79,172]. DNA damage response (DDR) is the mechanism maintaining genome stability through the coordination of checkpoint activation and DNA lesion repairs [69,173,174]. To ensure their physiological functions, embryonic SCs and adult SCs have a robust DDR capacity to remain their genome integrity and stability [79,175]. DDR is regulated by three apical kinases, ATM (ataxia telangiectasia-mutated), ATR (ataxia telangiectasia and Rad3-related), and DNA-PK (DNA-dependent protein kinase) kinases. Checkpoint activation halts cell proliferation, allowing lesions to be repaired [69,173,174]. The DDR process is compromised in aging stem cells, which is a likely mechanism underlying SC as an origin of cancer [79,175]. In PC, abnormalities in the ATM pathway were reported [176]. BMI1 is required for the maintenance of hematopoietic stem cells, neural stem cells [177,178,179,180,181,182], and intestinal stem cells [183,184]; BMI1 is also important in sustaining CSCs for multiple cancer types [185]. The upregulation of BMI1 occurs in PC [68]; the BMI1^+^ luminal cells are an origin of PC [67]. It has been recently reported that BMI1 reduces the functions of ATM and ATR during DDR, and thus contributes to genome instability [69,186,187,188]. In comparison to non-CSC PC cells, PCSCs display a robust DDR response to etoposide-induced double-strand DNA breaks (DSBs), which contributes to the resistance of PCSCs to DSB-associated toxicity [189]. Elevated DDR was also reported in patient-derived and cultured CSCs [79].

The ability to regenerate Lgr5^+^ CSCs from other colorectal cancer cells supports epigenetics being a major mechanism regulating CSC plasticity [168,190]. Epigenetic regulation can affect chromatin structure via histone methylation and ubiquitination [190]. EZH2 is the enzymatic subunit of the Polycomb-repressive complex 2 and mediates the trimethylation of histone H3 at lysine 27 (H3K27me3) [191]. The protein has a critical function in maintaining the CSCs of glioblastoma [192]. EZH2 expression is upregulated in PCSCs, and plays an essential role in PCSC growth [193]. PCSCs are regulated by a set of non-coding RNA molecules, MicroRNA-141, miR-34a, and miR-1991-3p [150,151,194]. Signatures of miRNA have been identified in PCSC and other CSC types [195]. From DU145 PC cells, PCSCs can be isolated in serum-free medium as spheres at the rate of 1.25%; when cultured in medium supplemented with 10% serum, it took 20 passages for the sphere-forming ability (which is indicative of stemness) of PCSCs to reduce to 2.2% [39], indicating the gradual loss of the PCSC-associated epigenetic pattern.

Recent developments reveal a critical role of partial EMT in regulating the lineage plasticity in multiple cancer types (see Section 4.1) [95]. Cancer cells with both epithelial and mesenchymal markers are plastic in driving distant metastasis through MET in HF-SCC [95,97,98]. In PC, macrometastasis in the lung expressed high levels of CKs and low levels of vimentin; the reverse profile of CK and vimentin was observed in micrometastasis [96], implying a contribution of partial EMT-associated plasticity in MET transition. The induction of EMT and prevention of its reversal to MET suppresses metastasis [196]. Overexpression of the paired-related homeobox transcription factor 1 (Prrx1) in Madin–Darby canine kidney (MDCK) cells induced EMT; ectopic Prrx1 expression in either MDCK or BT-549 cells prevented lung metastasis as a result of the inability of MET transition, while knockdown of the ectopically expressed Prrx1 enabled lung metastasis [196]. The intermediate metastable stage of EMT is critical in regulating trophoblast stem cells [197], supporting the important contributions of partial EMT in CSC plasticity. This concept is also supported by the partial EMT status detected in mesenchymal sarcomas [198]; this is because as a tumor with the mesenchymal origin, sarcomas may not need partial EMT to enhance its motility and invasion. In accordance with partial EMT regulating CSC plasticity, this EMT status likely contributes to the aggressiveness of sarcomas [198].

### 5.3. Molecular Basis of CSC Stemness and Plasticity

The current CSC model emphasizes the central importance of CSCs in cancer initiation and evolution. Thus, it remains critical to understand the molecular basis of reprogramming non-CSCs toward CSC. While current knowledge in this field remains limited, recent developments have shed light on this concept.

In PC, the inhibition of AR promotes PCSCs [199]. NANOG is a critical pluripotency reprogramming factor [200] that contributes to PCSCs [152]. Mutations in the *Speckie-type POZ protein (SPOP)* gene identify a subtype of human PC [201,202]; SPOP facilitates the homologous recombination repair of DSBs [203]; *SPOP* mutations are enriched with genomic alterations in the *IQGAP2* tumor suppressor gene in PC [204]. In line with these observations, SPOP was recently reported to suppress PCSCs through the degradation of NANOG [205,206]. Additionally, the signalings of Wnt, Sonic Hedgehog, Notch, Cav-1, and others in regulating PCSCs have been recently reviewed [207].

Genomic sequencing revealed a prevalence of mutations in *TP53* (53.3%) and *PTEN* (40.7%) in mCRPCs [208], suggesting an important interaction between these two tumor suppressors in suppressing the acquisition of a PCSC-like stage and/or the induction of the lineage plasticity of PCSCs during the progression of metastasis and CRPC. This concept is supported by the observations that PCs with the inactivation of both tumor suppressors are resistant to abiraterone and progress to neuroendocrine (NE) PC following abiraterone treatment in both mouse model and patients [209]. Loss of the *TP53* and *RB1* tumor suppressor genes are part of a signature event in NEPC development following anti-androgen treatment [210]. While PCs in prostate-specific *PTEN*^−/−^ mice are not generally metastatic, the co-knockout of *RB1* resulted in highly efficient (100%) metastasis to the lymph node, lung, and liver as well as bone (2/10) [211]. Triple knockout of *PTEN*, *RB1*, and *TP53* substantially enhanced NEPC progression following castration; the gene expression profile of these mouse NEPCs shares a similar profile with human NEPCs [211]. These observations are consistent with the association of the triple tumor suppressors with PC plasticity [212,213]. Furthermore, the NEPC lineage plasticity was also resulted from human primary prostate epithelial cells when engineered with AKT activation, RB1 knockdown, the expression of dominant-negative p53, MYC, and BCL2 [214]. Collectively, these new developments reveal a critical role of the tumor suppressor genes *TP53* and *RB1* in suppressing PCSC reprogramming. This process resembles the induction of iPSCs (inducible pluripotent stem cells), which is associated with DNA damage and the inhibition of p53 facilitates reprogramming toward iPSC [215].

**Figure 3:**
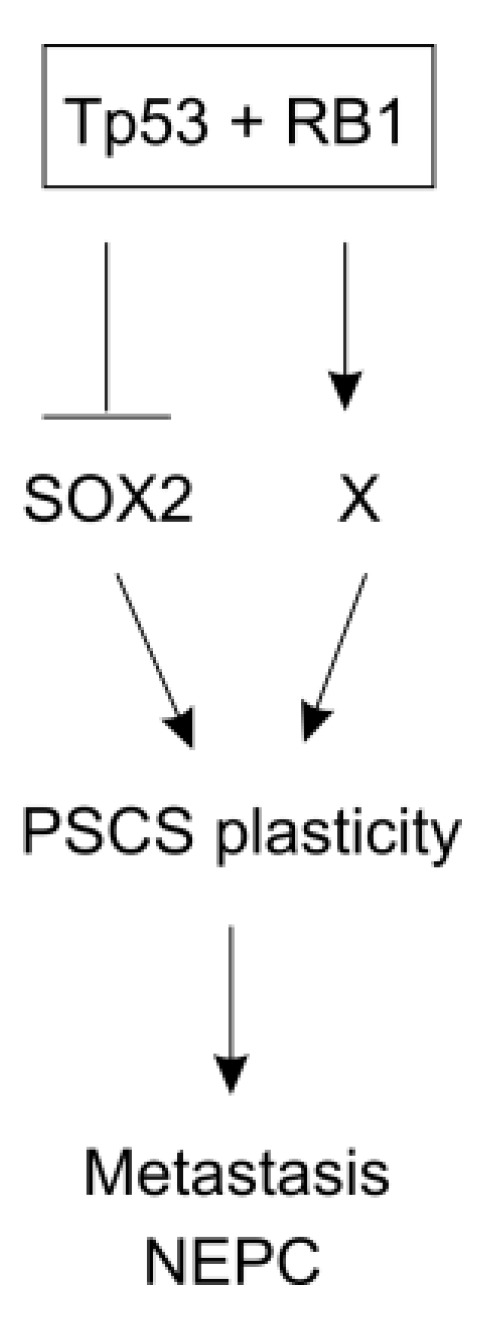
Current evidence reveals a critical role of TP53 together with RB1 in the suppression of SOX2 expression and affecting other events (X). SOX2 and X confer the cell plasticity of PCSCs, which promotes PC metastasis and induces neuroendocrine PC (NEPC) in response to anti-androgen therapies.

The co-inactivation of *PTEN*, *RB1*, and *TP53* upregulates SOX2 and EZH2 [211]. Through H3K27me3, EZH2 suppresses AR expression, which facilitates the reprogramming of PC to PCSCs [211]. Deficiencies in *TP53* and *RB1* induce lineage plasticity, which allows a switch from AR-dependent luminal cancer to AR-independent basal-like cancer following anti-androgen enzalutamide treatments [216]. This lineage switch is mediated by SOX2; the loss of *TP53* and *RB1* upregulate SOX2; the inhibition of SOX2 suppresses the lineage switch [216]. SOX2 was also reported to be responsible for a luminal-to-basal lineage switch in PCs produced by mouse BMI^+^ luminal cells with *PTEN*^−/−^ following castration [67,217]. Taken together, the evidence indicates an important contribution of SOX2 in the epigenetic reprogramming of PC cells toward PCSCs in response to anti-androgen therapies (Figure 3).

## 6. Conclusions

Overwhelming evidence supports the central role of CSCs in general and PCSCs in particular in cancer initiation, progression, and resistance to therapies. However, even with this optimistic opinion, direct evidence is lacking. In mouse PC models, lineage tracing with PTEN knockout as the oncogenic signal indicated PCSC as an origin of PC (Section 3), and suggested partial EMT as a factor inducing metastasis (Section 4); the inactivation of PTEN with the concurrent inactivation of TP53 and RB1 contributed to lineage plasticity leading to PCSCs (Section 5.3). A critical question is to what extent is this knowledge applicable to PC initiation and metastasis in patients? Is the occurrence of these oncogenic drivers the primary cause of PC or the outcomes of genome instability following PC tumorigenesis? In the latter scenario, these oncogenic drivers would be expected to promote rather than initiate PC. Nevertheless, accumulating evidence demonstrates the importance of PCSCs in PC tumorigenesis, both in initiation and progression.

It is an emerging concept that CSCs are a property of cancer instead of a specific group of cells that are intrinsically wired to be CSCs [168]. This discovery is generally consistent with our understanding of cancer being plastic, i.e., cancers evolve in response to toxicity caused by therapies. This observation suggests that targeting CSCs themselves might not be the perfect strategy, as these cells may not exist, and can be replenished upon environmental cues; instead, disrupting the pathways or targeting stromal events leading to the acquisition of lineage plasticity of CSCs could be more effective in controlling cancer growth and metastasis. The development of partial EMT in conferring lineage plasticity, particularly in CTCs, calls for modification regarding using EpCAM as the sole surface marker. In this regard, the use of EpCAM together with other mesenchymal markers should be explored.

## Figures and Tables

**Table 1:** PSCs are able to initiate PC.

Species	PSC ^1^	Oncogenic Signal ^2^	Tumor Model	Ref.
Human	Basal CD49f^hi^Trop2^hi^	AKT–ERG–AR	NSG s.c.—xenograft	[50]
Mouse	Basal Lin^−^Sca-1^+^CD49f^hi^	AKT1–AR	CB.17S^CID/SCID^ renal capsule	[55]
Mouse	Basal Lin^−^Sca-1^+^CD49f^hi^	PTEN knockout	SCID s.c.—xenograft	[56]
Mouse	Basal CK5^+^	PTEN knockout	Lineage-tracing	[60]
Mouse	Basal CK14^+^	PTEN knockout	Lineage-tracing	[63]
Mouse	Luminal CK8^+^	PTEN knockout	Lineage-tracing	[63]
Mouse	Luminal Nkx3.1expression	PTEN knockoutNkx3.1 knockout	Lineage-tracing ^3^	[65]
Mouse	Luminal BMI1^+^	PTEN knockout	Lineage-tracing	[67]
Mouse	Luminal LY6D^+^	PTEN knockout	Lineage-tracing—PIN lesion ^4^	[72]

^1^ The indicated cells with prostate stem cell (PCS) properties; ^2^ Oncogenic signals used to induce PC; ^3^ Reporter was driven under the Nkx3.1 tumor suppressor promoter; as a result, Nkx3.1 was knocked-out; ^4^ PINs were induced; in other models, PC was produced.

**Table 2:** CTCs contribute to metastasis.

Tumor ^1^	CTC ^2^	Outcome ^3^	Metastasis ^4^	Ref.
BC	EpCAM^+^CD44^+^CD47^+^MET^+^	OS ^6^	NSG mice; bone mets ^5^	[126]
BC	EpCAM^−^HER2^+^EGFR^+^HPSE^+^NOTCH1^+^	NA ^7^	Nude mice; lung met ^8^, brain met ^9^	[127]
CRC	CTC lines with CSC properties	NA ^7^	Nude mice; lung and liver met ^10^	[128]
PC	CK^+^Vimentin^+^CD45^−^	Met ^11^	NA ^7^	[100]

^1^ BC: breast cancer, CRC: colorectal cancer, PC: prostate cancer; ^2^ CTC types; ^3^ clinical outcome; ^4^ mouse model; ^5^ femoral medullar cavity implantation; ^6^ reductions in overall survival; ^7^ not available; ^8^ tail vein injection; ^9^ intracardiac injection; ^10^ spleen injection; ^11^ association with metastasis.
